# Supplementary material for: Highly efficient light management for perovskite solar cells
Source: Sci Rep. 2016 Jan 6;6:18922. doi: 10.1038/srep18922 (PMC4702131; doi:10.1038/srep18922)
Supplement: Supplementary Information [file srep18922-s1.doc]

**Supplementary Information**

Highly efficient light management for perovskite solar cells

Dong-Lin Wang^1^, Hui-Juan Cui^1^, Guo-Jiao Hou^2^, Zhen-Gang Zhu^2,1^, Qing-Bo Yan^3^ & Gang Su^1^*

^1^ School of Physics, University of Chinese Academy of Sciences, P. O. Box 4588, Beijing 100049, China.

^2^School of Electronic, Electrical and Communication Engineering, University of Chinese Academy of Sciences, Beijing 100049, China

^3^College of Materials Science and Opto-Electronic Technology, University of Chinese Academy of Sciences, Beijing 100049, China

*Correspondence and requests for materials should be addressed to G.S. (email: [gsu@ucas.ac.cn](mailto:gsu@ucas.ac.cn)).

**Supplementary Figures**


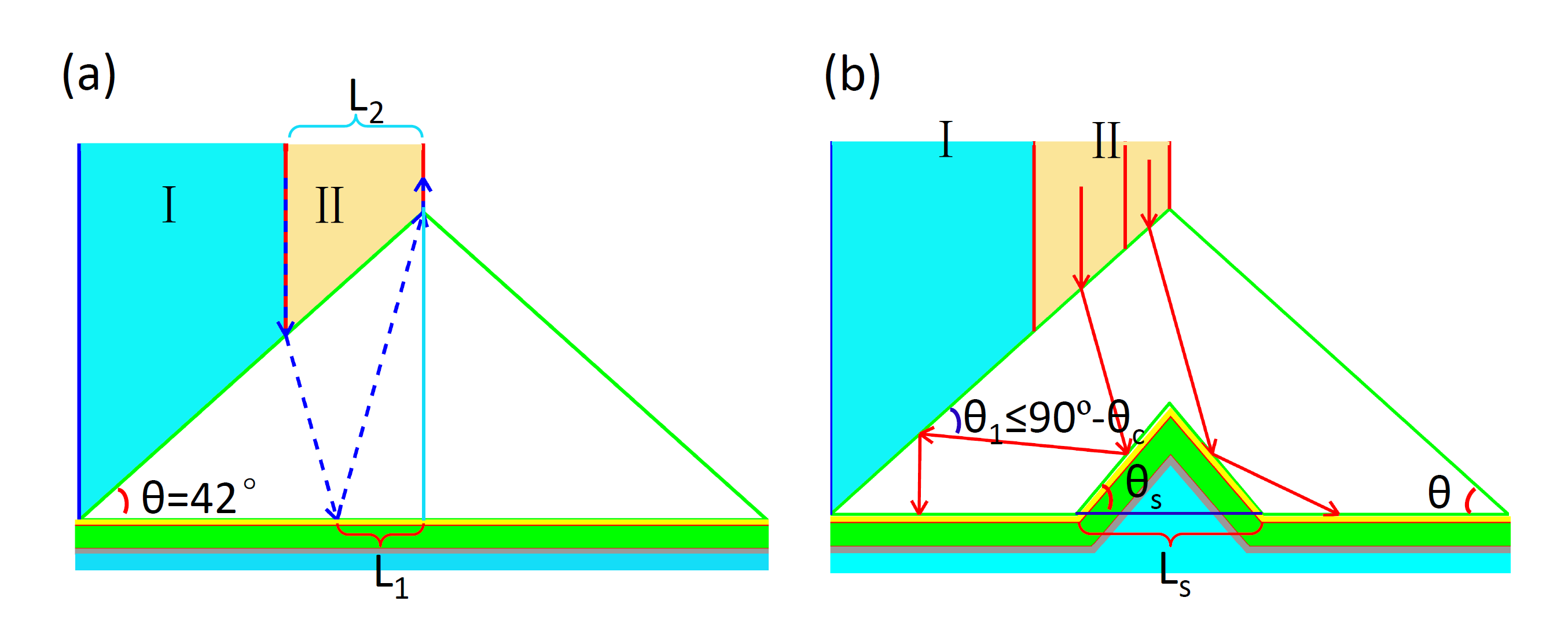


**Figure S1** Schematic of the light trapping induced by prism and slotted prism structures. (a) The light partitions in the perovskite solar cell with prism structure. (b) The representative light rays in the perovskite solar cell with the slotted prism structure.


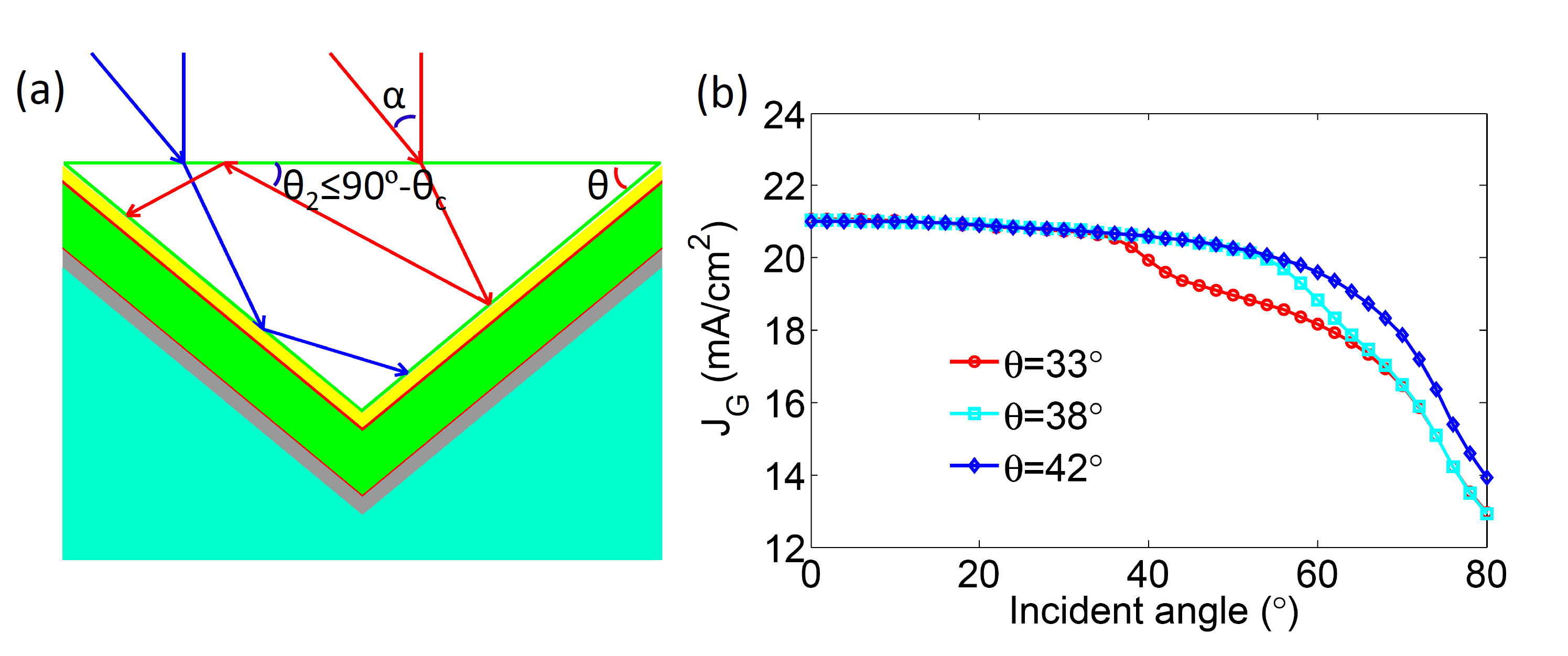


**Figure S2** Schematic of the light trapping induced by the inverted prism structure and the corresponding J_G_ of the designed perovskite solar cells. (a) The schematic of the light trapping in the perovskite solar cell with the inverted prism structure. (b) J_G_ of the perovskite solar cell with the inverted prism structure as function of the incident angles.


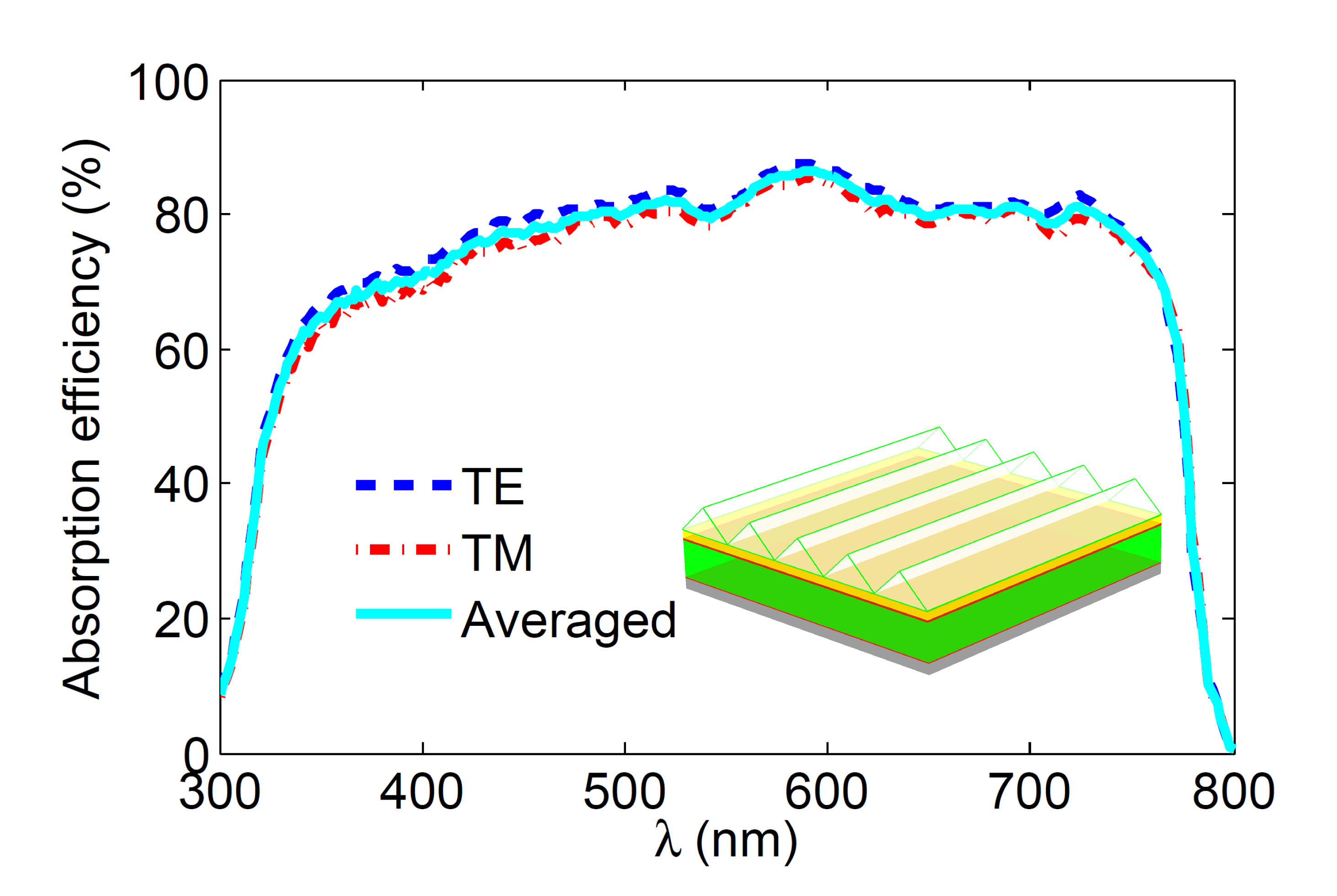


**Figure S3** Absorption efficiency in the perovskite layer for the TE and TM polarized incident light when the prism structured SiO_2_ structures are periodically arranged on the top surface of the perovskite solar cell.

**Supplementary Note1:**

**Design of the slotted prism based light trapping structure.** Proper designed prism structure can redirect the light direction to realize an efficient light trapping for the perovskite solar cell. For a prism with the base angle θ=42°, the multiple reflection of the incident light into the active layer can be realized when light comes fromⅠregion (see figure S1a). However, light loss is still hard to avoid for the light incident fromⅡregion. As shown in figure S1a, the size of theⅡregion is denoted as L_2_, and the critical size of the solar cell region that corresponds to L_2_ is marked as L_1_=L·tg(θ)·tg(θ-arcsin(sin(θ)/n_glass_))/2. To maximum the light trapping ability of the prism structure, we employ the slotted prism structures to reduce the light loss fromⅡregion. As shown in figure S1b, the bottom size of the slotted structure is denoted as L_s_, and the base angle is denoted as θ_s_. Here, L_s_ is set as slightly larger than 2L_1_ to minimize the influence of the light incident fromⅠregion. By employing the slotted prism structure, it is easy to realize double rejection of the incident light into the active layer when the light arrives at the right side of the slotted structure. Moreover, the total internal reflection at the prism side can reuse the escaped light from the left side of the slotted structure. To achieve this, a necessary condition that is θ_1_≤π/2-θ_c_ must be satisfied (see figure S1b), where θ_c_ is the critical angle for the total reflection of light from glass to air. By calculation, we can obtain θ_1_=π/2-2θ_s_+2θ-arcsin(sin(θ)/n_glass_), θ_c_≈41.5°, and also θ_s_≥49.5°. In this work, L=10μm, L_s_=2.75μm, and θ_s_=50° are considered in the simulation of the perovskite solar cell.

**Supplementary Note 2:**

**Design of the inverted prism based light trapping structure.** To enhance the serviceable angle of the perovskite solar cell, we employ the inverted prism structure to realize double rejection of the incident light into the active layer. As shown in figure S2a, we assume the light is oblique incident from the left side of the normal. It is clear that the double rejection of the incident light can be initiatively satisfied when the light arrives at the left side of the inverted prism. To reuse the reflected light that escapes from the right side of the inverted prism, we must ensure that the total reflection at the bottom of the prism is enabled. So, an indispensable condition that needs to be satisfied is θ_2_≤π/2-θ_c_ (see figure S2a). By calculation, we can obtain θ_2_=π/2-2θ+arcsin(sin(α)/n_glass_), subject to θ>arcsin(sin(α)/n_glass_), where α is the oblique angle of the light. The final solutions for above conditions give α<arcsin(n_glass_·sin(2θ-θ_c_)) for θ≤θ_c_ and α=90° for θ>θ_c_. In other words, the serviceable angle for realizing double injection of the light into the active layer can be adjusted by the base angle (θ) of the inverted prism. For example, the serviceable angle around 38° can be obtained by using the inverted prism with θ=33°, and such an angle about 58° can be achieved by the prism with θ=38°. Significantly, the serviceable angle for double rejection of the incident light can be improved to 90° by employing the inverted prism with θ≥42°. By implementing the simulation of the perovskite solar cell, the above principle can be verified by calculating the density of photo-generated current (J_G_) of the inverted prism at different oblique angles (shown in figure S2b). However, the serviceable angle for the prism with θ=42° only can be sustained within 60° due to the increased reflection occuring when the oblique angle is larger than the Brewster’s angle (~50° for the light injecting from air into glass).

**Supplementary Note 3:**

**Polarization dependence of the prism based light trapping structure.** The periodically arranged prism structured SiO_2_ also have the grating effect that can influence the light absorption in the solar cell. The grating can couple the incident light into the waveguide modes of the flat active layer by optical interference and diffraction effect. This coupling effect is more efficient under the weak absorption condition. As the perovskite has a strong capacity to absorb the light, the coupling effect of the grating can only slightly influence the light absorption in the solar cell. Moreover, the SiO_2_ based grating is transparent for all incident light, which further undermines the grating effect. So, the polarization dependence of the prism induced 1D grating is unobvious for the TE and TM polarized incident light. As shown in figure S3, the maximum difference of the absorption efficiency in the perovskite layer for TE and TM polarized incident light is only 5%, which is mainly induced by different transmissivity at the prism surface for above two polarized incident light.
